# Sorghum Seed Maturity Affects the Weight and Feeding Duration of Immature Corn Earworm, *Helicoverpa zea*, and Fall Armyworm, *Spodoptera frugiperda*, in the Laboratory

**DOI:** 10.1673/031.013.6701

**Published:** 2013-07-15

**Authors:** Alysha M. Soper, R. Jeff Whitworth, Brian P. McCornack

**Affiliations:** Department of Entomology, Kansas State University, 123 Waters Hall, Manhattan, KS 66502

**Keywords:** food consumption, grain sorghum, larval growth, phenology, *Sorghum bicolor*

## Abstract

Corn earworm, *Helicoverpa zea* Boddie (Lepidoptera: Noctuidae), and fall armyworm, *Spodoptera frugiperda* J.E. Smith, are occasional pests in sorghum, *Sorghum bicolor* L. Moench (Poales: Poaceae), and can be economically damaging when conditions are favorable. Despite the frequent occurrence of mixed-species infestations, the quantitative data necessary for developing yield loss relationships for *S. frugiperda* are not available. Although these species share similar biological characteristics, it is unknown whether their damage potentials in developing grain sorghum panicles are the same. Using no-choice feeding assays in the laboratory, this study examined larval growth and feeding duration for *H. zea* and *S. frugiperda* in the absence of competition. Each species responded positively when exposed to sorghum seed in the soft-dough stage, supporting evidence for the interactions between host-quality and larval growth and development. The results of this study also confirmed the suitability of using laboratory-reared *H. zea* to develop sorghum yield loss estimates in the field, and provided insights into the biological responses of *S. frugiperda* feeding on developing sorghum seed.

## Introduction

Sorghum, *Sorghum bicolor* L. Moench (Poales: Poaceae), is the fifth most produced cereal crop in the world and the third most important cultivated grain in the USA. Despite its environmental tolerances to drought (Rosenow et al. 1983, Rosenow and Clark 1995; Kebede et at. 2001) and stressful temperatures (Ougham and Stoddart 1986; Howarth 1989), sorghum remains susceptible to a wide array of insect pests. Yield losses caused by insects in sorghum costs American producers approximately $80 million annually (Wilde 2006). Pest management programs throughout USA sorghum production regions typically focus on the sorghum midge, *Contarinia sorghicola*, greenbug, *Schizaphis graminum*, and several panicle-feeding caterpillars, including sorghum webworm, *Celama sorghiella* Riley, corn earworm, *Helicoverpa zea* Boddie (Lepidoptera: Noctuidae), and fall armyworm, *Spodoptera frugiperda* J.E. Smith (Young and Teetes 1977; Wilde 2006). Under favorable conditions, *H. zea* and *S. frugiperda* populations can cause significant yield reduction by feeding directly on developing sorghum seeds (Young and Teetes 1977). *H. zea* and *S. frugiperda* are the most frequently observed lepidopterous pests that feed on whorl-stage sorghum as well as on developing seeds (Chamberlain and All 1991).

Mixed-species infestations of *H. zea* and *S. frugiperda* in panicles occur throughout the United States sorghum producing regions (Teetes and Pendletom 2000). Colonization of these species is significantly aided by adult flight capabilities and favorable weather fronts that drive moths northward from source populations in southern Texas or Mexico (Sandstrom et al. 2007; Westbrook 2008).

Although both species are polyphagous, earlyseason migrants prefer to lay eggs in whorlstage or silking corn (Capinera 2005, 2007). Larvae of both species are often associated with plant blossoms, buds, and fruits. Consequently, as silking corn begins to senesce in Kansas, USA, adjacent, lateplanted sorghum fields in the half-bloom through hard-dough stages are readily infested when moths disperse away from maturing corn (Vanderlip 1993; Stichler et al. 1997; Sloderbeck et al. 2008). Planting sorghum early to avoid damaging larval infestations is not always feasible or effective, depending on the timing of moth immigration events.

Interactions between timing of larval infestations and sorghum growth stage (Vanderlip 1993; Stichler et al. 1997) can further complicate treatment decisions for sorghum producers. For example, both *H. zea* and *S. frugiperda* must complete six larval stages before burrowing into the soil to pupate (Capinera 2005, 2007), making host availability essential to successive generations. This is particularly true for *S. frugiperda*, which has host-specific strains (Pashley 1986) that are morphologically similar but genetically different (Lu and Adang 1996; McMichael et al. 1999; Nagoshi and Meagher 2003; Nagoshi et al. 2006). More specifically, *S. frugiperda* shows host-plant preference for either corn and sorghum (corn strain) or rice and forage grasses (rice stain) (Pogue 2002). During peak moth activity and subsequent larval development, sorghum panicles are also progressing through three reproductive stages, which include the flowering, soft-dough, and hard-dough stages (Vanderlip 1993; Stichler et al. 1997). Soft-dough stage sorghum is most vulnerable to third to sixth instar *H. zea* feeding, which accounts for 95% of the damage observed in the field (Kinzer and Henderson 1968). Kinzer and Henderson (1968) also found that first and second *H. zea* instars preferred flowering sorghum. Consequently, sorghum phenology plays a key role in determining yield loss relationships. In addition, insecticide treatments are often warranted in late-planted sorghum fields that are at greater risk to infestation, especially when natural enemies are in low abundance (Wiseman 1985; Sloderbeck et al. 2008); the effect of sorghum phenology on *S. frugiperda* growth and feeding habits is not known.

In Kansas sorghum, current management guidelines for headworm infestations urge growers to balance market values with treatment costs prior to making a treatment decision (Michaud et al. 2010), which is based on previous bioeconomic models developed by Buckley and Burkhardt (1962) and Kinzer and Henderson (1968). Individual *H. zea* larvae cause approximately 6% grain damage per larva (Buckley and Burkhardt 1962; Kinzer and Henderson 1968), resulting in an economic threshold of 1 to 2 larvae per panicle (Teetes and Pendleton 2000). Despite the frequent occurrence of mixed-species infestations, quantitative data necessary for developing yield loss relationships are not available for *S. frugiperda* (Buntin 1986; Chamberlain and All 1991), yet the same economic injury level established for *H. zea* (2 to 3 larvae per panicle; Knutson and Cronholm 2007) is applied to *S. frugiperda* infestations (Martin et al. 1980; Teetes and Pendleton 2000; Michaud et al. 2010). As a result, independent management guidelines do not currently exist for *S. frugiperda* in developing sorghum panicles. While it is generally known that both species feed directly on developing sorghum grain (Buntin 1986; Teetes and Pendleton 2000), quantitative data showing the impact of panicle feeding exists only for *H. zea* (Buckley and Burkhardt 1962; Kinzer and Henderson 1968; Teetes and Wiseman 1979). Major assumptions in these guidelines are that the damage capacity for the two species and their sorghum stage preferences are equal. While both species share similar biological characteristics, such as developmental times, reproductive capacity, and dispersal rates (Sparks 1979; Chamberlain and All 1991; Capinera 2005, 2007; Westbrook 2008;), differences in larval growth and development at critical sorghum development stages are not known.

Assumptions made in the development of pest management strategies can greatly influence the ability of growers and managers to make correct decisions. Consequently, validating such assumptions leads to improved decisionmaking, which can lead to increased yields and high-value integrated pest management programs. In this regard, it is important to learn how these two species respond to host developmental stages and whether they represent an equivalent threat to maturing sorghum grain. Therefore, the objectives of this study were to: 1) compare larval weights and feeding duration between *H. zea* and *S. frugiperda* feeding on key sorghum reproductive stages, 2) determine differences in larval growth between field and laboratory reared populations, and 3) identify the suitability of using laboratory-reared larvae as an experimental proxy for field populations when estimating sorghum yield loss.

## Materials and Methods

To control for other factors affecting the feeding behavior of larvae, such as species competition or food preference, laboratory experiments were conducted on individual larvae of *H. zea* and *S. frugiperda* using nochoice feeding assays containing a single sorghum spikelet. Experimental units or the no-choice feeding arenas were made of a thinwalled, clear-plastic tube, 17 cm × 5.6 cm diameter (Cleartec Packaging, www.cleartecpackaging.com), with a tight-fitting end-cap at the base. The top of each arena was covered with white, no-see-um mesh (Quest Outfitters, www.questoutfitters.com) that allowed for air passage, and secured with a rubber band.

*H. zea* and *S. frugiperda* larvae were obtained from 20-year-old laboratory colonies, neither of which had been amended with field collected specimens for at least 11 years (Benzon Research, Inc., www.benzonresearch.com). A mitochondrial DNA analysis of adult *S. frugiperda* (n = 18) collected from this laboratory colony demonstrated that 94% of the individuals from this mixed colony were predominately of the corn strain (data not shown). For the duration of the experiment, each larva remained in the same feeding arena and received a newly excised sorghum spikelet of the same developmental stage every 24 hr. Dead larvae were replaced with new individuals from the same cohort, which were concurrently maintained on an artificial corn-based diet (Benzon Research Inc.). New larvae were recorded as separate replicates in the analysis (Kinzer and Henderson 1968). Larval developmental stages were not recorded during this study, and none of the individuals used were maintained through to pupation.

Sorghum spikelets were cut from field-collected panicles during a two-week period from early to mid August in 2010. Every 3 to 4 days, panicles of the appropriate developmental stage (flowering, soft-dough, or hard-dough; Vanderlip et al. 1993) were collected from production fields in Geary, McPherson, Riley, and Washington Counties, KS. Varied planting dates created differences in sorghum developmental stages. Specifically, hard-dough panicles were collected from early-planted fields, while flowering and soft-dough stages were readily found in later-planted fields; sorghum spikelets of commercially available varieties (i.e., Pioneer 84G62, 84P74, 85G03, 85Y40) were divided among *H. zea* and *S. frugiperda* treatments. Extra panicles were collected and stored in a refrigerator (3° C ± 1° C) up to 3 days prior to larval exposure.

### Larval weight and feeding duration

A 2 × 3 factorial design was used to assess larval growth on sorghum spikelets at different phenological stages. Each species-sorghum treatment combination was confined using the individual feeding arenas (n = 180) described previously. Specifically, main effects consisted of species (*H. zea* and *S. frugiperda*) and sorghum growth stage (flowering, soft-dough, and hard-dough) with duration of exposure (days) to each sorghum stage as a repeated measure. Due to the feeding capacity and associated damage potential of late-instars (third through sixth instars; Kinzer and Henderson 1968), feeding assays were initiated using third instars. All larvae were weighed (g) every 24 hr using an analytical balance (Denver Instrument, Pinnacle Series P-114, www.denverinstrumentusa.com; error ± 0.0001). Change in larval weight was used as an indirect measure of sorghum consumption and direct measure of larval growth. Feeding arenas were arranged in a completely randomized design on a laboratory bench and kept at room temperature (∼22° C) with a photoperiod of 16:8 L:D.

### Source colony validation

**Figure 1.**
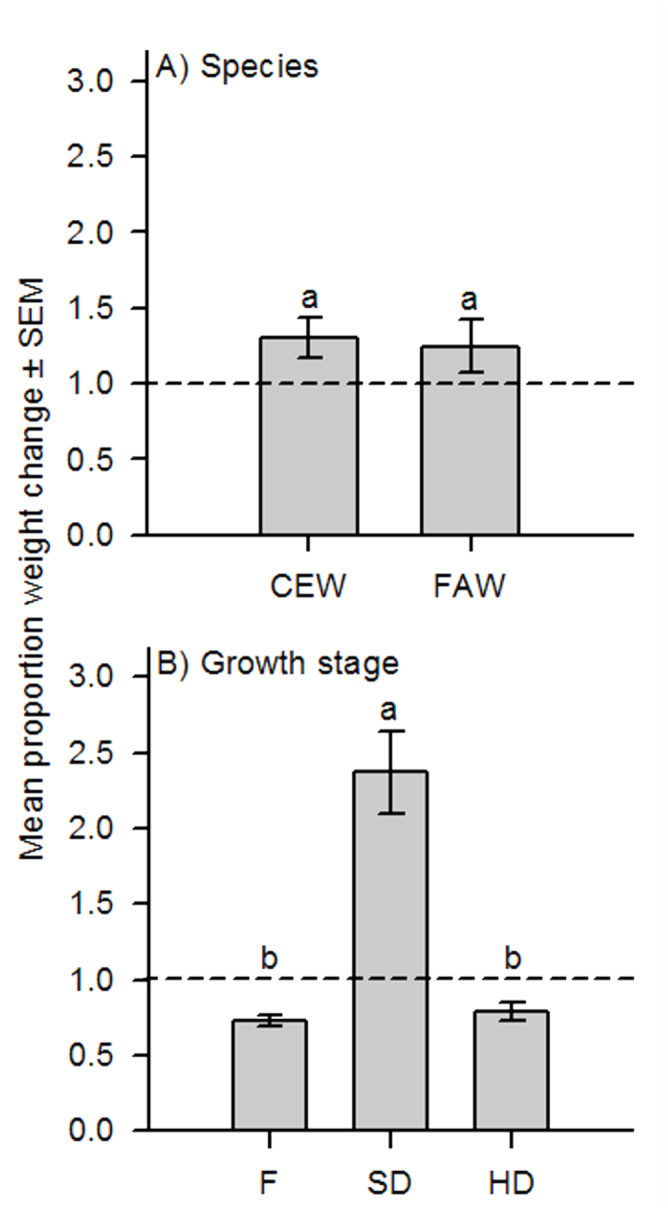
The A) species and B) sorghum stage (flowering = F, soft-dough = SD, and hard-dough = HD stages; Vanderlip et al. 1993) effects on the mean proportion weight change ± SEM of corn earworm (CEW), *Helicoverpa zea*, (n = 172) and fall armyworm (FAW), *Spodoptera frugiperdz*, (n = 110) larvae used in a repeated measures laboratory study. Weight measured in g (± 0.0001). Mean proportion weight change values above or below 1.0 represent weight gains or losses, respectively. Bars with the same letter are not significantly different at α = 0.05. High quality figures are available online.

**Figure 2.**
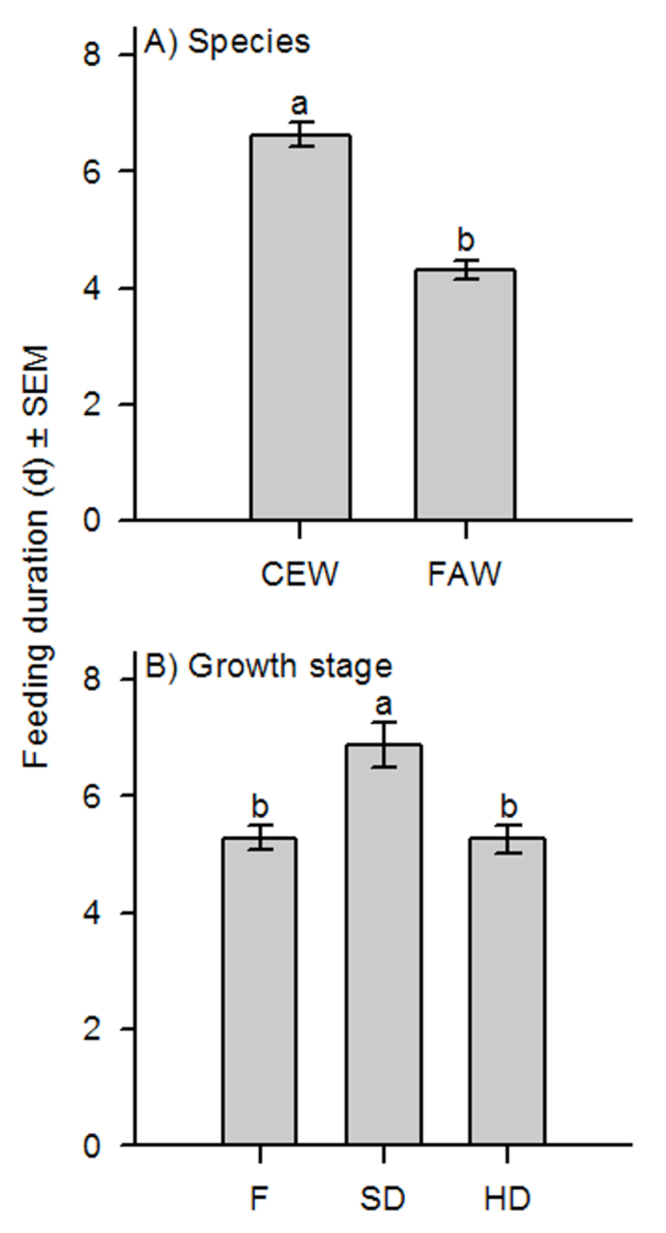
Mean feeding duration (days) ± SEM for corn earworm (CEW) and fall armyworm (FAW) larvae feeding on sorghum at three sorghum growth stages: flowering = F, soft-dough = SD, and hard-dough = HD. High quality figures are available online.

To identify whether laboratory findings could be applied to larval populations in the field, differences in larval weights were tested between laboratory-reared and field-collected source populations. Specifically, two populations of *H. zea* (field-collected and labreared corn earworm, hereafter referred to as “field CEW” and “lab CEW”, respectively) and a lab-reared population of *S. frugiperda* (hereafter “lab FAW”) were compared. Field-collected *S. frugiperda* larvae were not available for this experiment. For the wild population, 30 third-instar *H. zea* were collected from a production sorghum field in the soft-dough stage (var. Pioneer 84G62) at the Ashland Bottoms Research Farm near Manhattan, KS, on 6 August 2010. No-choice feeding assays were conducted for each of the three source populations tested (n = 30 per treatment), and larvae were allowed to feed for 5 days. Based on results from the previous experiment (see results; Figure 1, 2), third instars were only fed with field-collected sorghum spikelets in the soft-dough stage.

In a concurrent field study, the differences in source colonies were examined using exclusion cages (n = 10 per source colony type), which enclosed a single sorghum panicle (var. Pioneer 84G62). Enclosure of the panicle prevented any seed damage by other arthropods and vertebrates (e.g., birds), while protecting experimental larvae from natural enemies. Exclusion cages consisted of white, no-see-um mesh (Quest Outfitters) with zippered tops (23 cm diameter, 71 cm long). Zippers provided easy access to the panicle after cage installation. The base of each exclusion cage was secured using 15.2 cm zip-ties (Gardner Bender, www.gardnerbender.com) just below the peduncle. To allow free-movement of larvae within the cage, cylindrical supports made of 14-gauge, galvanized steel wire rope (Impex Systems Group, Inc., Miami, FL) were added, keeping the mesh from resting on the panicle.

All panicles were sampled prior to cage installation using the beat-bucket method (Merchant and Teetes 1992) to avoid selection of naturally infested panicles. Exclusion cages were infested with 10 third-instars from the lab and field CEW source colonies. Larvae were placed on panicles using fine, camel-hair paint brushes (#1). All cages were left in the field for the duration of seed head maturity and remained on each sorghum panicle through harvest in late September 2010. Following harvest, damaged seeds on individual sorghum panicles were counted and used as a measure of larval feeding. Control panicles, caged at the time of infestation, were used to determine the level of environmental damage (seed counts described below) experienced by treatment panicles over the course of the experiment.

Larval growth or survivorship was not directly measured in the field, so yield loss was used as an indirect measure to differentiate population performance. To accomplish this, damaged seeds were categorized and counted as undeveloped seed, fungus-infected seed, or larva-consumed seed, as previously described by Buckley and Burkhardt (1962). Undeveloped seeds can be the result of larval feeding on and clipping the palea and lemma structures during the early flowering stage, which ultimately prevents embryo development. Consequently, environmental factors, like water stress (Fenner 1992; Rosenow and Clark 1995), can also prevent seeds from forming or filling properly. Saprophytic “field fungi” and some *Fusarium* spp. will often invade exposed germplasm after larval feeding and cause the fungus-infected seeds to appear dark and moldy (Cunfer 2008). Finally, feeding damage includes seeds with exposed white germplasm, which is a direct result of larval consumption. Once damaged seeds had been counted, the entire seed head was threshed. Undamaged seed was easily separated during the threshing process and the remaining seed was weighed (g). Proportion yield loss was calculated as:


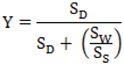


where Y equals the proportion yield loss for an individual sorghum panicle; SD represents the total number of damaged seeds across all damage categories; S_w_ is the total weight (g) of threshed seed per panicle; S_s_ equals the mean seed size (g/seed), which was estimated using mean 100-count seed weights (3 per head); and S_w_/S_s_ represents the estimated number of undamaged seeds in the panicle.

### Statistical analysis

To standardize the larval growth response, daily proportion weight change was calculated for all individuals used in the study. Specifically, the end weight (g) of larva for each 24 hr period was divided by the initial larval weight (g) at the time of first exposure to a treatment. To account for within-subject, time-dependent correlations associated with taking multiple measurements on the same individuals (Wang and Goonewardene 2004; Littell et al. 2006) a mixed model approach was implemented using a repeated measures analysis to test differences in larval weight change (PROC MIXED, SAS Institute 2002). The fixed main effects in the model included species (*H. zea, S. frugiperda*), stage (flowering, soft-dough, and hard-dough sorghum), and exposure (total number of days a larva was exposed to treatment). Starting weight (g) was a covariate to account for the influence of larval size on growth rates (Abrams et al. 1996). Simple effects tests were explored for significant interactions by slicing main effects (Littell et al. 2006). Differences in treatment groups were determined using generalized least squares with a Tukey-Kramer multiple comparisons adjustment. Because the response variable for feeding duration was the number of days larvae survived and fed on a given sorghum reproductive stage, a generalized linear model was used to test for differences in the main effects of species and sorghum stage (PROC GLM, SAS Institute 2002).

For the source colony experiment, weight response for each of the three colonies examined (field CEW, lab CEW, and lab FAW) was calculated and analyzed in a second repeated measures model. With the exclusion of sorghum stage, all explanatory variables were the same. In the field study, pre-existing seed damage was corrected for in the exclusion cages by subtracting the proportion yield loss observed in control cages from the damage calculated in treatment panicles. Differences in the mean proportion yield loss between *H. zea* colonies (field CEW versus lab CEW) were estimated using a two-sample *t*-test (PROC TTEST, SAS Institute 2002). The *F*-ratio was used to test fit at a significance level of α = 0.05.

## Results

### Larval weight and feeding duration

**Figure 3.**
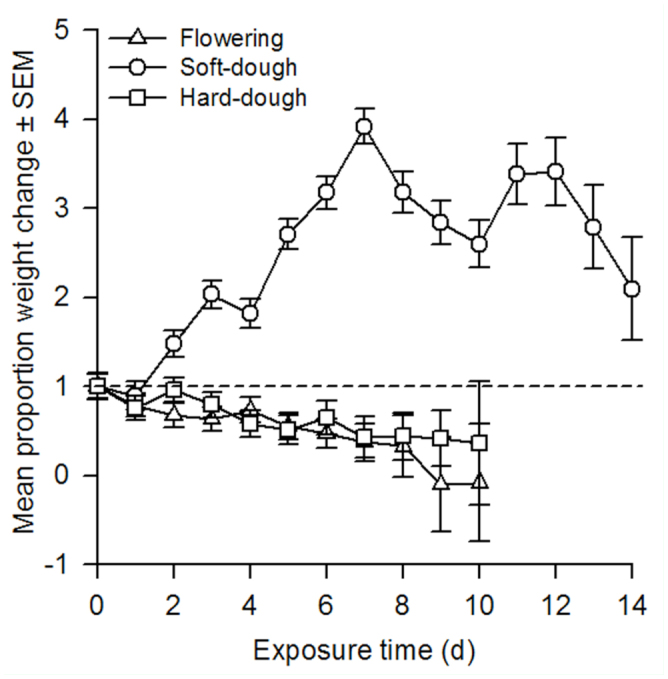
Mean proportion weight change ± SEM representing larval growth of the pooled response of corn earworm and fall armyworm over time (days) when feeding on three reproductive stages of sorghum (flowering, soft-dough, and hard-dough) in a repeated measures laboratory study. Mean proportion weight change values above or below 1.0 represent weight gains or losses, respectively. High quality figures are available online.

The effect of sorghum reproductive stage on changes in proportion weight and feeding duration was consistent between both species tested (Table 1). In general, *H. zea* initial weights ranged from 0.0014 to 0.5845 g, and final weights from 0.0001 to 0.4388 g, while *S. frugiperda* initial weights ranged from 0.0001 to 0.0008 g, and final weights from 0.0001 to 0.0303 g. Because the actual initial and final weights of each species were quite variable, proportion weight changes were used to account for these differences. Specifically, *H. zea* (n = 172) and *S. frugiperda* (n = 110) proportion weight gains remained unchanged when averaged across all sorghum reproductive stages (Figure 1A). Contrastingly, larval feeding duration was different between species, such that *H. zea* larvae fed and survived for approximately 2 days longer than *S. frugiperda* (Figure 2A). In general, changes in larval weight were influenced by the length of exposure (days) to a given sorghum reproductive stage regardless of species (Figure 3). Larval starting weights (g) did not significantly influence larval growth (Table 1); therefore, this explanatory variable was excluded as a covariate in the final model.

**Table 1.**
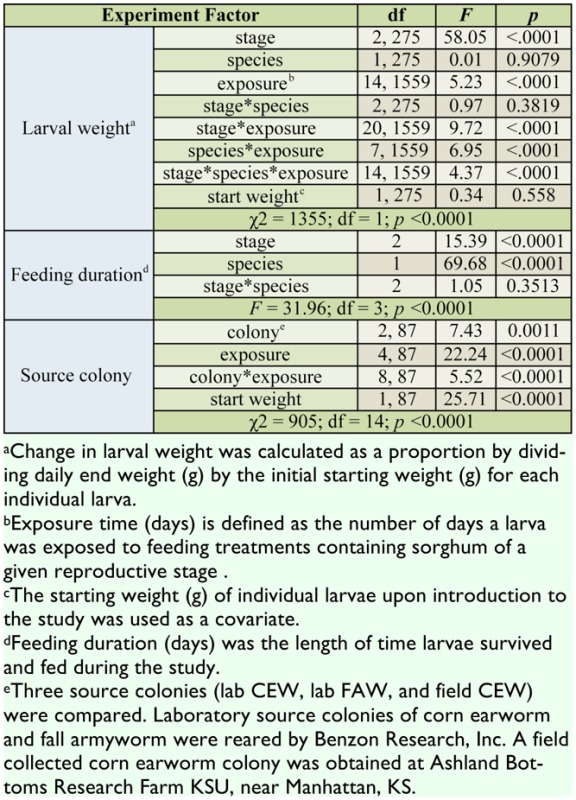
Analysis of variance results for the effects of sorghum reproductive stage (flowering = F, soft-dough = SD, and hard-dough = HD) on the mean proportion weight change and feeding duration (days) of corn earworm (*Helicoverpa zea*) and fall armyworm (*Spodoptera frugiperda*) larvae in the laboratory and field.

There was no significant interaction between sorghum reproductive stage and species in terms of larval weight change or feeding duration (Table 1). Although the proportion weight change for *H. zea* and *S. frugiperda* was the same at each sorghum stage, weight gain was 1.6 times greater overall in soft-dough than either the flowering or hard-dough stages (Figure 1B). Similarly, larvae of both species fed for nearly 2 days longer when exposed to soft-dough sorghum rather than flowering or hard-dough stages (Figure 2B). Changes in larval weights were significantly affected by the interaction between sorghum reproductive stage and exposure time (days). Slicing for sorghum stage showed that this main effect did not significantly influence larval weight change until ≥ 2 days after initial exposure (Ps < 0.0001). Conversely, sorghum stage significantly influenced larval weight change at flowering (*F* = 2.64; df = 7, 1559; *p* = 0.0104), soft-dough (*F* = 23.91; df = 7, 1559; *p* < 0.0001), and hard-dough stages (*F* = 6.99; df = 7, 1559; *p* < 0.0001) when sliced across exposure time. The proportion weight change of larvae tested was influenced by the interaction between species and exposure time (days). Slicing for effects showed that species, *H. zea* (*F* = 5.80; df = 10, 1559; *p* <0.0001) and *S. frugiperda* (*F* = 7.34; df = 7, 1559; *p* < 0.0001), significantly affected the proportion weight change of larvae tested. Exposure time had an inconsistent effect on larval weight change. A three-way interaction between stage, species, and exposure time was also observed. When sliced across stage and species, the effect of exposure time to treatment was not different for *H. zea* feeding on either the flowering or hard-dough stages, but was significant for both species feeding on soft-dough (*P*s <0.0001). Exposure was also significantly reduced in all *S. frugiperda* treatments (*P*s <0.008). Keeping the effects of stage and exposure time fixed, species response did not change over time for any sorghum stage (*P*s >0.05). Slicing the interaction by species and exposure time showed that the effect of sorghum stage did not become significant until 2–3 days following initial exposure for *S. frugiperda* and *H. zea*, respectively (*P*s < 0.007).

### Source colony validation

**Figure 4.**
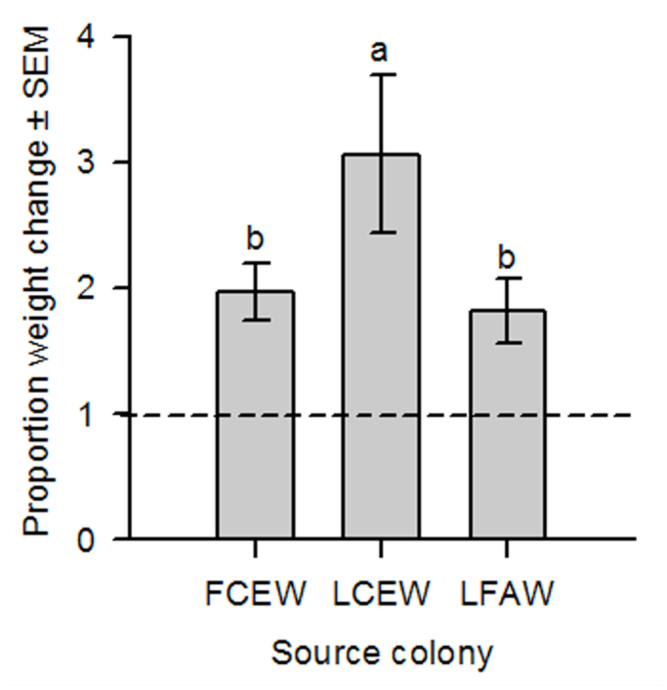
Mean proportion weight change ± SEM for larvae from three source colonies: “FCEW” = field-collected corn earworm, *Helicoverpa zea*, (n = 29); “LCEW” = laboratory-reared corn earworm, (n = 30); and “LFAW” = laboratory-reared fall armyworm, *Spodoptera frugiperda*, (n = 32), feeding on soft-dough stage sorghum for 5 days in a repeated measures laboratory study. Mean proportion weight change values above or below 1.0 represent weight gains or losses, respectively. Bars with the same letter are not significantly different at α = 0.05. High quality figures are available online.

When larvae were exposed to soft-dough sorghum, proportion weight change was significantly different between larval sources; lab CEW individuals gained 35–40% more weight than either the field CEW or lab FAW larvae, respectively (Table 1; Figure 4). Feeding duration was not significantly different between colonies (*F* = 1.27; df = 2; *p* = 0.285). Exposure time to soft-dough sorghum had a significant effect on the proportion larval weight change when averaged across source colony, increasing 2.6 times over the 5-day exposure period. Larval starting weight (g) significantly influenced larval growth and was included as a covariate in the final model (Table 1). There was also a significant interaction between source colony and exposure time on larval weights. When sliced, this interaction showed that both colony and exposure time increased proportion weight change after 1 day (*P*s <0.006).

Environmental damage observed in control panicles in the field was 28%. Artificial infestation of field CEW and lab CEW colonies in field cages showed no significant differences in mean proportion yield loss (0.30 and 0.31, respectively) due to larval feeding damage (t = -0.13, df = 18; *p* = 0.8996).

## Discussion

This study provides insight into the effects of host phenology on the success of noctuid pest larvae in developing sorghum panicles. Specifically, this research demonstrated that both *H. zea* and *S. frugiperda* (corn-strain) responded positively to soft-dough stage sorghum in terms of weight gain and feeding duration. This result not only supports previous work conducted by Kinzer and Henderson (1968), which showed that *H. zea* of the third through sixth instar preferred to feed on soft-dough sorghum in the laboratory, but also provides evidence that *S. frugiperda* responded similarly to developing sorghum seed. Additionally, *S. frugiperda* from a 94% corn-stain colony responded in equal magnitude to4 *H. zea* in terms of weight gain, regardless of sorghum stage. These results provide the first quantitative evidence that *S. frugiperda* and *H. zea* may be equivalent threats to sorghum seed yields, which has important implications for assessing field infestations under current management guidelines.

Several studies investigating *S. frugiperda* in the field indicated that crop-specific damage depended on the host-strain dominating the population (Pahley 1985; Meagher and Nagoshi 2004). Given that over 90% of the *S. frugiperda* colony used in this study originated from corn-strain populations, it is likely that the observed response to sorghum as a food source was representative of a population expected to infest sorghum under field conditions. In this study, the mixed-strain colony of *S. frugiperda* did not survive as long as *H. zea* feeding on sorghum under laboratory conditions, but they were still capable of gaining 10% more weight than *H. zea* on the flowering stage and nearly 30% more on the hard-dough stage. However, both species experienced optimized growth and survival on soft-dough stage sorghum. In combination with the results published by Kinzer and Henderson (1968) and Wiseman et al. (1986) showing that neonate *H. zea* and *S. frugiperda* preferred flowering stage sorghum, these data demonstrate that host crop phenology plays an important role in influencing the feeding behavior of these developing insects. For example, third instars were able to subsist on unfavorable sorghum stages in the laboratory (i.e., flowering and hard-dough), but began feeding for longer periods of time and gaining weight only on soft-dough seed. Therefore, field-planted sorghum may be most vulnerable to yield loss by infestations of either *H. zea* or *S. frugiperda* (corn-strain) when panicles are in the early seed-fill or soft-dough stage.

As potential threats to sorghum yield loss, *H. zea* and *S. frugiperda* larvae in the third through sixth instar may be equally destructive. It was found that not only did the effects of proportion weight change and feeding duration change equally for *H. zea* and *S. frugiperda* (additive effects) in response to sorghum stage, but also there was no significant difference in larval weight change between species across sorghum stages. Slicing significant interaction terms showed that the species did differ in proportion weight change and feeding duration only when having fed on soft-dough sorghum for at least 2 days. Despite the soft-dough being the optimum host stage for each species, *S. frugiperda* fed on it for significantly fewer days than *H. zea* in the laboratory. In the field, larvae would be exposed to the continuous physiological development of sorghum seed rather than the static feeding scenario explored in this laboratory experiment. It is most likely that weight changes and feeding duration in the field would more closely reflect the equivalent larval responses observed in the laboratory when averaged across all three sorghum developmental stages.

This experiment tested for differences in species response within a given sorghum treatment group such that all larvae fed on sorghum spikelets excised from panicles of the same variety, stage, and condition. The overall effect of spikelet excision from sorghum panicles on larval behavioral or physiological responses is unknown. However, excision of fresh spikelets from field-collected sorghum panicles has been used as a method to examine larval feeding preference, development, and host plant resistance under laboratory conditions (Kinzer and Henderson 1968; Diawara et al 1991a, b). While it has been shown that tannin content within various developmental stages of sorghum seed does not affect *S. frugiperda* growth on meridic diet (Wiseman et al. 1986), high concentrations of acid detergent fiber and tannin in hard-dough sorghum seed has been shown to correlate with *S. frugiperda* resistance (Diawara et al 1991b). Furthermore, a number of environmental factors, such as temperature, water availability, and light, may dramatically influence the nutrient content and potential quality of developing seed in the field (Fenner 1992). For example, high temperatures and drought can increase seed protein content and may alter the balance of fatty acids (Rosenow and Clark 1995), perhaps enough to influence insect attraction to the seed as a food source. The effect of nutrient content in maturing sorghum seeds on host quality for either *H. zea* or *S. frugiperda* development has not been investigated and may be a focus for future work.

Laboratory feeding assays containing only soft-dough sorghum spikelets showed that the lab CEW colony grew significantly more in terms of weight gain than either the lab FAW or field CEW colonies. The difference in the lab-reared colonies confirmed the species differences seen in the larval weight and feeding duration study. A similar growth differential occurred between the *H. zea* populations; lab CEW grew significantly more than field CEW. The lack in field CEW response could have been an artifact of the wild population being ill-adapted to a transition to laboratory conditions or to moderate handling.

While proportion yield loss in the field study cannot directly be compared to the weight response of larvae in the laboratory, both response variables can be interpreted as indirect measures of larval consumption or damage potential for CEW populations in sorghum. In the field, no differences were apparent between the two CEW colonies in terms of damage potential. Although these studies were conducted using larvae maintained in a colony for over 20 years, this comparison study suggests that use of the labreared CEW colony was valid in the field and could be used in the laboratory to generate estimates for conservative treatment recommendations.

Sorghum is an increasingly important field crop in the USA, and with ongoing advancements in sorghum breeding programs, this cereal is well-placed for shaping the future of food in many parts of the world (KGSPA 2011). In order for sorghum integrated pest management programs to be successful, growers must be provided with updated and accurate management recommendations. Although guidelines have been in place for the management of *H. zea* populations in sorghum, this research confirmed that sorghum reproductive phenology played an important role in determining *H. zea* survival and damage potential, and provided the first documentation for it in *S. frugiperda*. Although this research demonstrated that the assumption of equivalence of *H. zea* and *S. frugiperda* larvae feeding in sorghum panicles may be correct, it also confirmed that sorghum was most vulnerable to yield loss by *H. zea* and *S. frugiperda* during the soft-dough stage. Scouting during the early seed fill will be critical to making an accurate management decision for either species independently or in combination. This study also confirmed the applicability of these results to *H. zea* populations in the field, and provided insights into the biological responses corn-strain *S. frugiperda* feeding has on developing sorghum seed in the laboratory. Future field studies should investigate the use of currently implemented management recommendations, which are based on 50-year estimates for *H. zea* yield loss potentials (Buckley and Burkhardt 1962), and test for species differences with *S. frugiperda* in the field.

## Abbreviations

CEW: corn earworm
FAW: fall armyworm
